# Use and Abuse of Poultices

**Published:** 1867-10

**Authors:** Benj. Richardson


### ARTICLE VII.

### *Use and Abuse of Poultices.*

By Dr. Benj. Richardson.

In his lectures recently delivered at the College of Physicians, Dr. Richardson made the following remarks on the subject of poultices :

The application of moist heat in the form of poultice to suppurating parts requires, I think, remodeling, in order that it may be placed on a true scientific basis. I am afraid that the common recommendation, "You must put on a poultice," is too often among us all an easy way of doing something about which we are not quite sure, and concerning which it were too much trouble to think long.

From what I have recently observed, I fear that mischief is often done by a poultice which might well be avoided. The people have always a view, that a poultice is applied to "draw," as they say—a term in truth which, though very unsophisticated, is in a sense a good term, for it means what it says. The question for us is, whether it be sound practice to carry out as a general rule the "drawing" process, either by fomentation or by poultice.

When a part is disposed to suppurate, the first step in the series of changes is an increased flow of blood through the capillary surface, followed by obstruction, and thereupon by an excess of sensible heat derived from the friction that is set up. Then follows transudation of liquor sanguinis into the connective tissue, and its transformation, under the influence of heat, into what is called purulent fluid. When to the part in this state we apply moist heat, we quicken suppuration, mainly by upholding the temperature; and at the same time, we secure the transference of water from the moist surface into the fluids of the inflamed part, by which tension of tissues is produced, and in the end yielding of tissue at the weakest point.

When the suppurating surface is circumscribed, the rapid induction of the process may be attained with little injury; when the surface is large, and when the exuded fluid is thrown into loose structures where it can burrow readily, the practice I think, cannot be good to extend the mischief. Hence, in the treatment of carbuncle and phlegmonous erysipelas, it cannot, I opine, be sound practice in the early stage to apply moist heat. Experience also, not less than principle, warrants this conclusion.

In cases of carbuncle especially, I have of late altogether avoided the application of moist heat in the early stage, I feel assured, with good results.

But when in the course of local disease suppuration is actively established, and is naturally circumscribed; when the increased temperature of the part has fallen to or below the natural temperature—then the value of moist heat comes on with full force; then the tension which is exerted determines the escape of fluid at the weakest point of the surrounding tissue, and when the fluid escapes or is liberated by the knife, the escape for a long period is aided by the application of moist heat.

The continued application of moist heat for a long time after the escape of purulent fluid is again, I conceive, indifferent practice. It sustains discharge; it sets up unhealthy decomposition of fluids; it produces a thickened, soddened condition of skin, most favorable to the production of sinus; and it retards recovery. When a surface is freely open and suppurating, dry, and not moist heat is the remedy. We are in want in these cases of a simple invention; we require something which we can apply as readily as a poultice, which shall keep up the temperature of the part, and at the same time take up moisture, and gently dessicate without injuring the tissues.—*British Medical Journal.*

---

